# A CARE-compliant case report: total pancreatectomy and total gastrectomy to treat pancreatic ductal adenocarcinoma

**DOI:** 10.1097/MD.0000000000018151

**Published:** 2019-11-22

**Authors:** Yanghui Wen, Junhao Tu, Xiaofeng Xue, Weiqiang Shi, Lei Qin, Haixin Qian, Yinkai Xu, Xiaolan Xu

**Affiliations:** a Department of General Surgery, The First Affiliated Hospital of Soochow University; b Department of General Surgery, Suzhou Wuzhong People's Hospital, Jiangsu Province, China; c Department of Pathology, The First Affiliated Hospital of Soochow University; d Department of Gastroenterology, Xiangcheng People's Hospital, Suzhou, China.

**Keywords:** pancreatic ductal adenocarcinoma, total gastrectomy, total pancreatectomy

## Abstract

**Rationale:**

Total pancreatectomy (TP) is performed in cases of multifocal and large invasive tumors of the pancreas, and is associated with high rates of mortality and morbidity. Previously, the limitations and unsatisfactory effect of this surgery rendered it rarely performed; however, with improvements in surgical techniques and blood sugar management, TP is now more frequently performed. TP has a similar long-term survival rate as that for pancreatoduodenectomy (PD). However, the application of TP plus total gastrectomy (TG) for the treatment of invasive pancreatic ductal adenocarcinoma has not been reported previously.

**Patient concerns:**

The patient was a 64-year-old man with epigastric discomfort. Physical examination showed a hard mass. Preoperative computed tomography and magnetic resonance imaging revealed a solid mass located in the pancreatic body and involving the portal vein and stomach.

**Diagnosis:**

Pancreatic cancer.

**Interventions:**

The patient was treated with TP combined with TG and portal vein reconstruction.

**Outcomes:**

The patient had a smooth post-operative recovery but, regretfully, developed metastases 2 months after discharge.

**Lessons:**

Considering the poor outcome of the present case, the validity of the operation should be reevaluated. Although a single case does not elicit a convincing conclusion, the current case might serve as a warning against performing a similar surgery.

## 1 Introduction

Total pancreatectomy (TP) is adopted in patients to extend oncological radicality and reduce morbidity and mortality due to pancreatic fistulae.^[1–3]^ However, the uncontrolled glycemic index and exocrine deficiency caused by TP are major issues in clinical practice. Although simultaneous islet autotransplantation (IATx) is commonly applied to patients with TP, the safety of its application in the setting of pancreatic malignancy still requires further investigation.^[4]^ Due to improvements in surgical techniques and the use of synthetic insulin, the TP-related mortality has decreased and the patient quality of life has improved.^[1,5–8]^ Furthermore, there is no evidence (in terms of glycated hemoglobin levels, hypoglycemia, and ketoacidosis) that the controlled glycemic index in patients who undergo TP is worse than that in those who undergo pancreatoduodenectomy (PD). Additionally, TP is currently considered an alternative and viable treatment for patients with large and multifocal tumors.^[9,10]^

To the best of our knowledge, the application of TP plus total gastrectomy (TG) for the treatment of pancreatic cancer has not yet been reported. Pancreatic neck tumors involve the pancreatic head and body, and in such cases, TP is the best choice. Additionally, portal vein (PV) involvement in pancreatic neck tumors is not rare. Thus, resection and reconstruction of the PV are commonly performed in TP cases.^[11–14]^

Herein, we present a case of a large adenocarcinoma involving the PV and treated by TP with TG and PV reconstruction. Although the tumor contacted the superior mesenteric vein with an irregular contour, a complete resection and vein reconstruction was allowed; in the NCCN guidelines, this is defined as “borderline resectable”. A previous retrospective review of 160 patients with borderline resectable tumors reported a median survival duration of 40 months in patients with a successful surgical intervention (R0) and 15 months in patients who did not undergo any interventions.^[15]^ Another study of 129 patients reported median survival durations of 33 and 12 months in patients who did not undergo resection, respectively. Thus, considering that surgical resection is the only potential curative treatment to improve the survival rate, such patients could be selected for surgery to determine the likelihood of acquiring negative resection margins (R0).^[16]^

## 2 Consent

The ethics committee of the Medical College of Soochow University approved this report, and informed written consent for publication of this case report and the accompanying images was obtained from the patient's son.

## 3 Case study

A 64-year-old man was admitted to the Department of General Surgery because of a huge tumor located in the pancreatic body. He did not have any special medical history. A physical examination revealed a palpable mass, measuring 4 to 5 cm in diameter, in the epigastric region. The mass was hard and had a poorly differentiated boundary. Routine laboratory test values were basically normal, with the exception of the hemoglobin level (108 g/L; normal range, 130–175 g/L), prealbumin level (123.9 mg/L; normal range, 200–400 mg/L, and fibrinogen level (6.71 g/L; normal range, 1.8–3.5 g/L). Tumor biomarker levels were also mainly within the normal range, with the exception of cancer antigen 12 to 5 (95.30 U/ml; normal range, 0–35 U/L).

**Figure 1:**
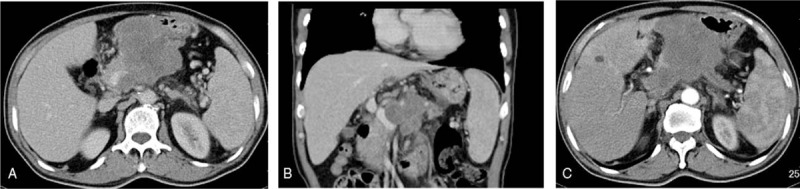
Preoperative CT images of the patient. A. CT revealed a large mass located at the pancreatic body. B. The portal vein was invaded by the tumor. C. The main pancreatic duct was dilated to 6.5 mm. CT = computed tomography.

**Figure 2:**
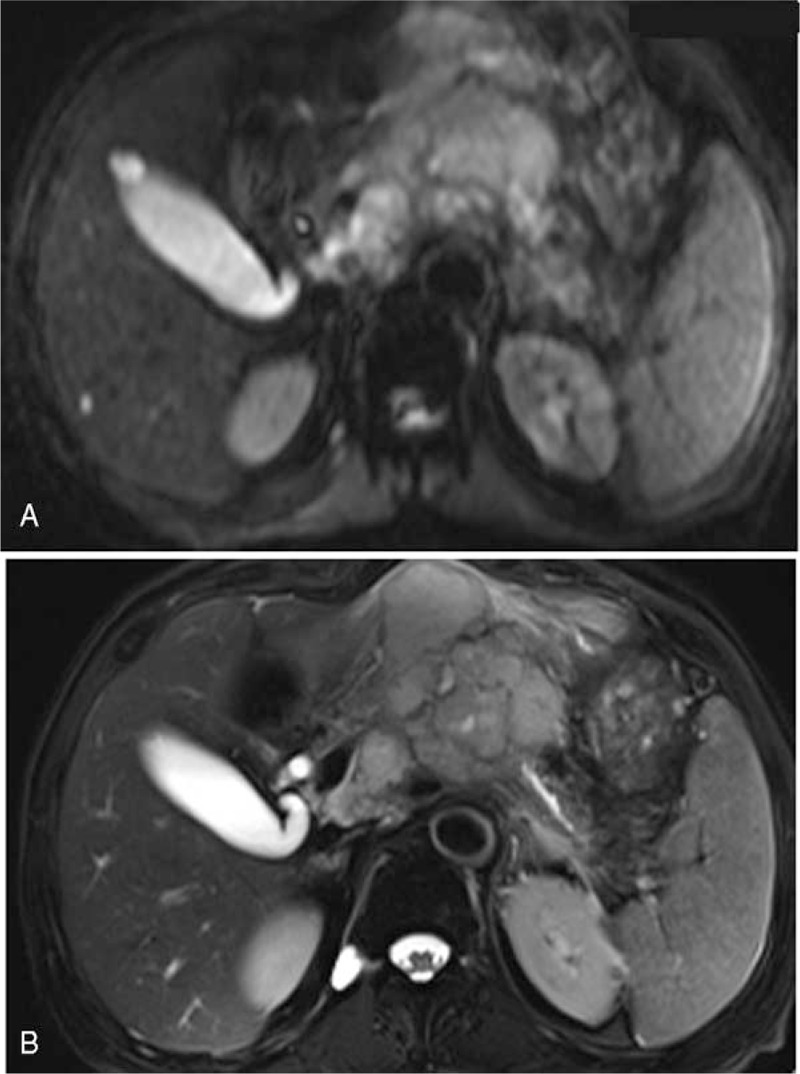
Preoperative magnetic resonance images of the patient. A. The lesion in the pancreatic body has high signal intensity on diffusion-weighted imaging. B. On T2-weighted images, the mass exhibits high signal intensity.

Preoperative contrast-enhanced computed tomography (CT) revealed a solid mass located in the pancreatic body, with an unclear boundary between the PV and stomach, and weak and non-uniform enhancement (Fig. 1A, B). The distal pancreatic duct was dilated to 6.5 mm (normal range, 2–4 mm; Fig. 1C). A dilated liver bile duct was not observed inside or outside; however, multiple retroperitoneal lymphadenopathies were revealed on CT (Fig. 1A). As shown in Figure 2A, the lesion had high signal intensity on diffusion-weighted imaging (DWI). The mass also exhibited high signal intensity on T2-weighted images (Fig. 2B). Based on the results of these examinations, a pre-operative diagnosis of pancreatic adenocarcinoma was established.

Considering the condition of the tumor, TP with TG and a potential PV reconstruction was decided. Once his basic condition was evaluated and contraindications were excluded, a surgery was planned. A median incision was first performed. The liver, abdominal cavity, and pelvic cavity were then carefully searched; no metastases were detected. However, a tumor (5 × 4 × 4 cm in size) was located in the pancreatic neck and body, invading the gastric corpus anteriorly, pancreatic head rightly, portal vein posteriorly. An enlarged lymph node was palpated in the ligamentum hepatoduodenale. These findings, along with the results of the preoperative examinations, further indicated that PD was not feasible. Therefore, TP, TG, splenectomy, and PV reconstruction, followed by esophagojejunostomy and choledochojejunostomy, were subsequently performed.

**Figure 3:**
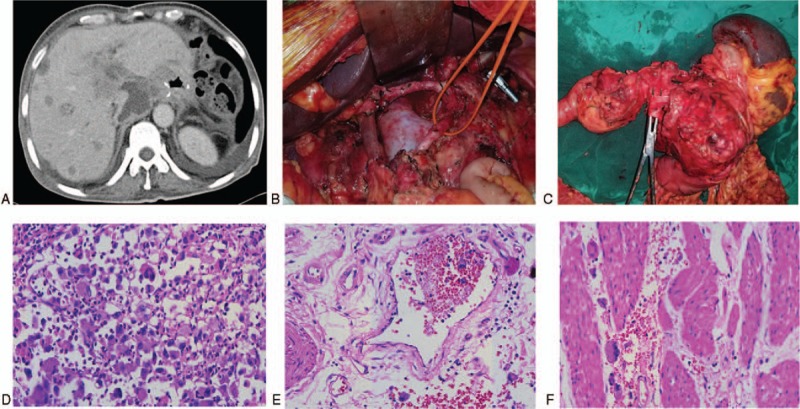
A. CT images at 2 months postoperatively suggest multiple hepatic metastases. B. The surgical field after removing the specimen. C. The specimen comprised the total pancreas, total stomach, and spleen. D. Poorly differentiated pancreatic ductal adenocarcinoma. E. Vessel carcinoma embolus revealed by hematoxylin and eosin staining. F. Tumor invasion of the gastric parietal muscularis.

The surgery comprised the following steps:

1. mobilization of the pancreatic head and duodenum;
2. division of the bile duct and mobilization of the pancreatic neck;
3. removal of a segment of the PV at the confluence, reconstructed with 5–0 prolene using end-to-end anastomosis (Fig. 3B);
4. mobilization of the body and tail of the pancreas and spleen, followed by removal of the total pancreas, stomach, and spleen (Fig. 3C); and
5. esophagojejunostomy and choledochojejunostomy. The operative time was 7 hours and blood loss was 1000 ml.

The tumor, accompanied with vessel carcinoma embolus, was diagnosed as poorly differentiated pancreatic ductal adenocarcinoma (PDAC) using hematoxylin and eosin staining (Fig. 3D and E). The gastric parietal muscularis was infiltrated with cancer cells and the margins of the stomach (Fig. 3F), bile duct, and pancreas were tumor-free. Among the 24 lymph nodes removed, 4 were identified as metastases. Thus, the tumor was classified as stage III (T3N2M0), in accordance with the 8th edition of the AJCC cancer staging for pancreatic cancer. The immunohistochemical tests were positive for CK, CK7, and Ki-67 (approximately 40%), and negative for CD56, CgA, Syn, and Her2.

The patient transitioned to the intensive care unit for 1 day and then returned to the general ward. He had an uneventful recovery, with 5 days for defecation and 4 days for diet. Pleural effusion occurred postoperatively, which was treated with thoracentesis. Insulin and pancreatic enzymes were used as endocrine and exocrine replacements. During the first week, parenteral nutrition was adopted for nutritional support and 30 to 32 units of insulin were required to control the blood glucose level. Once parenteral nutrition was terminated, 8 units each of long-acting and short-acting insulin were required to control the glucose level, and the patient achieved ideal glycemic control. However, he was re-admitted 2 months later for abdominal pain, fever, weight loss, and poor appetite. A whole-abdomen enhanced CT scan revealed multiple hepatic metastases (Fig. 3A).

## 4 Discussion

TP was first performed by Rockey in 1943, resulting in death due to bile leakage.^[17]^ The initial aim of TP was the avoidance of pancreatic fistulae, which were the most common cause of postoperative death; however, TP failed to reduce the mortality rate despite the extended pancreatectomy.^[18]^ Although TP was previously associated with high rates of morbidity and mortality,^[19,20]^ the results have been encouraging in recent years.^[7]^ Specifically, the morbidity and mortality of TP have significantly decreased as a result of enhanced surgical techniques and the support of insulin and digestive enzymes.^[1,8]^ Several studies have reported no difference in mortality between PD and TP.^[5,21,22]^

TP is the best choice for multifocal and large invasive tumors, including intraductal papillary mucinous neoplasm of the pancreas, islet cell neoplasms, and neuroendocrine tumors. Based on the 2017 NCCN guidelines for pancreatic adenocarcinomas, we judged the tumor in the present case as borderline resectable, and TP was indicated due to the tumor's large size and intensive invasion. In the present case, the tumor had invaded the gastric corpus and PV, forcing us to additionally perform TG and PV reconstruction. PV invasion was previously regarded as a preclusive factor for surgical intervention.^[23]^ However, recent studies have indicated that resection of the PV is safe, with no association between PV resection and the survival rate.^[14,24]^ Nevertheless, reports regarding TP plus TG are lacking. Based on the final pathological examination, the tumor in the present case was diagnosed as PDAC (the most common type of pancreatic cancer), which should only be treated by surgery. However, only 20% of patients treated by surgery reach a 5-year survival.^[17]^

A retrospective analysis of 616 patients with pancreatic cancer who underwent surgical resection was previously conducted. Univariate and multivariate models indicated that tumor diameter (*P* = .0008), negative margins (*P* = .008), negative nodes (*P* = .002), moderate tumor differentiation (*P* = .004), and adjuvant therapy (*P* = .0001) are predictors of the survival and recurrence rates in patients who undergo pancreatectomy; however, in multivariate models, negative nodes was not a significant favorable factor (*P* > .05).^[25]^ Another study of 226 patients treated between 1990 and 2002 suggested that tumor size (*P* = .03), differentiation (*P* = .02), R0 resection (*P* = .03), and absence of postoperative complications (*P* = .009) were predictors of long-term survival.^[26]^ In the present case, the large size and poor differentiation of the tumor indicated a poor prognosis.

Clinical trials of various types of chemotherapy for stage IV pancreatic cancers have been extensively conducted. In general, the median overall survival (OS) of patients with advanced pancreatic cancer ranged 5.9 to 12.2 months in these various trails, with the median survival of patients who received chemotherapy prolonged to some extent.^[27–31]^ On the other hand, there are many retrospective reviews of cases defined as borderline resectable; a successful resection (R0) was achieved in approximately one third of patients. These previous studies also demonstrated the safety and efficacy of neoadjuvant chemoradiation.^[15,32–35]^ Generally, the median OS of patients with borderline resectable pancreatic cancer who underwent resection ranged 16.4 to 40 months. Moreover, in all of these studies, patients with borderline resectable pancreatic cancer who achieved a successful resection had a better median OS compared to that in those with stage IV pancreatic cancer treated only by chemotherapy (*P* < .05). Pathological results in the present case indicated a negative margin, which confirmed the success of the surgery.

Surgical treatments adopted for borderline resectable pancreatic cancer include TP, PD, and distal pancreatic resection. While PD comprises the major part of the surgery, it is reasonable to compare the short-term and long-term outcomes of patients who undergo TP with those who undergo PD. A previous study of 100 patients who underwent TP and 1286 who underwent PD demonstrated that those who underwent TP have worse outcomes than do those who undergo PD. The median OS was 12.6 months for TP and 21.0 months for PD (*P* < .05). In contrast, the long-term outcomes of TP and PD were comparable (3-year survival, 27.5% vs 26.8%; 5-year survival, 18.9% vs 18.5%; both *P* > .05). The study also found that preoperative mortality decreased over time as the number of TP cases increased, suggesting that TP is safe and effective for appropriate patients, acquiring a prolongation of survival, when performed in experienced centers.^[36]^ The application of TP plus TG for the treatment of pancreatic malignancy has not been previously reported. A previous study suggested that TG with distal pancreatectomy and splenectomy for the treatment of gastric cancer does not improve the outcome of patients and is associated with more severe complications compared to those in patients who do not undergo an additional pancreatectomy.^[37]^ In the present case, since lesions in the liver were not found on preoperative CT, the tumor cells had most likely spread from the tumor thrombus of the PV. Considering the poor outcome of this patient, the validity of our surgery should be reassessed.

TP followed by IATx might obviously ameliorate the need for glucose control.^[38]^ However, the application of IATx in pancreatic malignancy still remains controversial and should be considered with extreme caution.^[39,40]^ Pancreatic malignancy remains an exclusion criterion at all centers due to a fear of diffusing the carcinoma cells.^[41]^ Therefore, we did not offer IATx to the patient for the large invasive tumor, which may increase the chance of disease recurrence. The patient was informed of the large risk of tumor recurrence and complications owing to the late stage of the tumor and the potential need for major surgery. Although he had a smooth and encouraging recovery, with a stable blood sugar level, his physical condition gradually deteriorated after discharge. One month postoperatively, the patient was supported by total parenteral nutrition. Two months postoperatively, the patient regrettably developed metastases. Considering the potential benefit of adjuvant chemotherapy for PDAC,^[42,43]^ we recommended it to the patient, but he refused the treatment.

A major limitation in the present case is the absence of neoadjuvant chemotherapy and radiotherapy. Previous studies have indicated that neoadjuvant chemoradiation is a potential method to promote resectability and reduce local recurrence.^[44,45]^ Several studies have also proposed that neoadjuvant therapy offers the possibility of downstaging, thus enhancing the likelihood of a R0 resection. However the rate of downstaging was not significant, ranging 3.4% to 11%.^[46,47]^ Although neoadjuvant therapy is currently suggested as the initial therapy for borderline resectable cases, with the advantages of better patient selection for surgery, early micrometastasis intervention, and the possibility of acquiring some degree of downstaging and increasing the likelihood of a R0 resection. Therefore, neoadjuvant therapy should be considered in such cases. However, the current patient refused neoadjuvant treatment because of a fear of tumor progression. Additionally, preoperative staging laparoscopy was reasonable in the present case, considering the large size of the tumor.

In conclusion, the present case of an expanded surgery resulted in poor outcomes. Thus, the decision to do an extension of TP, such as TG, should be made with extreme caution, even with negative margins. Although a single case precludes a survival analysis and does not provide a final conclusion, the current case might serve as a warning against performing a similar surgery.

## Acknowledgments

The authors gratefully acknowledge the patient's family for their support in this report.

## Author contributions

**Conceptualization:** Xiaofeng Xue.

**Data curation:** Junhao Tu, Weiqiang Shi, Haixin Qian.

**Formal analysis:** Lei Qin.

**Writing – original draft:** Yanghui Wen.

**Writing – review & editing:** Yanghui Wen, Xiaolan Xu, Yinkai Xu.
